# Producing fluorescent plants to lure and trap insect pests

**DOI:** 10.1111/pbi.13887

**Published:** 2022-07-24

**Authors:** Qinglin Peng, Jing Zhao, Siya Xiang, Jiajia Li, Chaochao He, Xingting Huang, ManZhu Bao, Jihua Wang, Genfa Zhu, Robert M. Larkin, Hong Luo, Guogui Ning

**Affiliations:** ^1^ Key laboratory of Horticultural Plant Biology, Ministry of Education Huazhong Agricultural University Wuhan China; ^2^ College of Plant Science and Technology Huazhong Agricultural University Wuhan China; ^3^ Flower Research Institute of Yunnan Academy of Agricultural Sciences National Engineering Research Center For Ornamental Horticulture Kunming China; ^4^ Guangdong Key Laboratory of Ornamental Plant Germplasm Innovation and Utilization Institute of Environmental Horticulture, Guangdong Academy of Agricultural Sciences Guangzhou China; ^5^ Department of Genetics and Biochemistry Clemson University Clemson SC USA

**Keywords:** fluorescence, green fluorescent protein, transplastomic tobacco, lure and trap, insects, pests

Light traps make important contributions to integrated pest management strategies by helping to reduce the impact of humanity on the environment. There is considerable interest in developing light sources that utilize less energy and that are more eco‐friendly. Insects can perceive wavelengths of light that range from 650 to 300 nanometres. Recently, ultraviolet lights have been used as an effective tool for attracting insect pests (Cook *et al*., 2007). When GFP is excited at 475 nm light, the subsequent emission maximum is 503 nm (Verkhusha and Lukyanov, 2004). It was suggested the fluorescence from chemical compounds attracts insects to carnivorous plants (Kurup *et al*., 2013). To date, no reports have tested whether fluorescence influences insect behaviour. There are no reports on whether it is feasible to use the fluorescence emitted by GFP to develop pest control technology. *Spodoptera litura* (Fabricius) has phototactic responses and damages lots of vegetable and field crops (Yang *et al*., 2012). In our present study, firstly varied transgenic tobaccos were developed by chloroplast and nuclear transformation (Figure S1) and fluorescein isothiocyanate (FITC) treated tobacco was also integrated. Finally, we performed a series of experiments to evaluate interactions between insects and various types of tobacco.

We produced different fluorescent tobacco lines. We observed that substantial GFP fluorescence was emitted from transplastomic tobacco lines possibly because the large number of chloroplasts in leaf cells facilitate high‐level expression of exogenous protein. Indeed, most leaves emitted green light and single cells strongly fluoresced (Figure 1d–f and d'–f'), which was not the case for the transgenic tobacco plants that harboured a nuclear‐localized transgene that expresses GFP (Figure 1a–c and a'–c'). The wavelengths of light emitted from leaves of the transplastomic tobacco that were excited at 475 nm were different from the leaves of wild‐type tobacco (Figure 1f,f').

**Figure 1:**
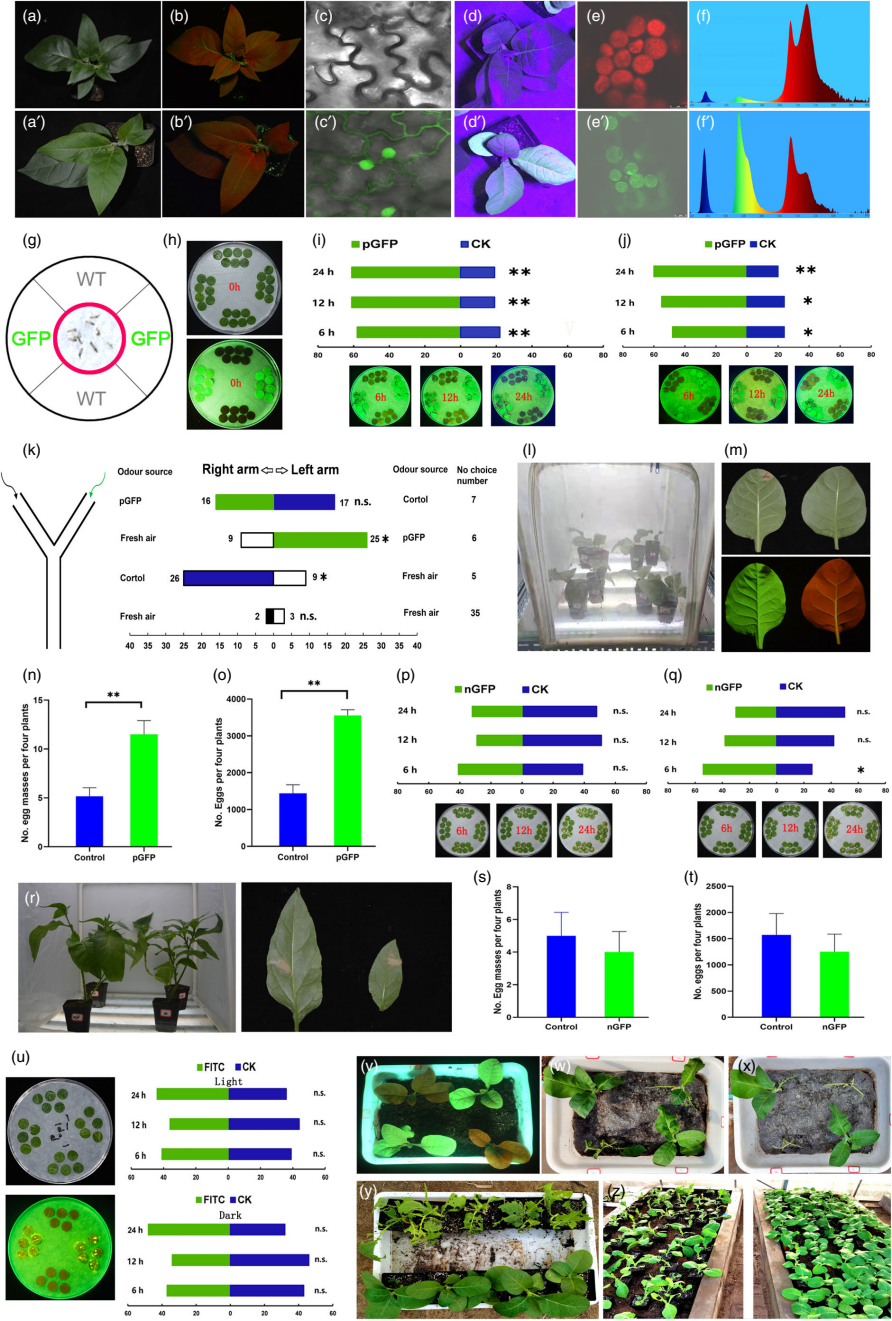
Producing fluorescent plants to lure and trap insect pests. a–c: Wild type tobacco (a–c) and transgenic tobacco (a'–c') expressing GFP from the nuclear genome (nGFP). Photo acquired in white light (a), photo acquired in 475 nm light (b) and it is absence of green fluorescence in the cells of control tobacco (c). d–f: Wild type tobacco (d–f) and transplastomic tobacco plants (d'–f') emitting high intensity fluorescence with an emission maximum of approximately 500 nm in the cells and leaves (pGFP). g–j: Feeding preference of *S. litura* larvae for leaf discs exposed to light and in the dark in a two‐choice assay. k: Numbers of *S. litura* larvae attracted in the odour selectivity testing using glass Y‐tube olfactometer. l–o: Oviposition preference of *S. litura* adults for the transplastomic tobacco plants harbouring GFP. p–q: Feeding preference of *S. litura* larvae for leaf discs of nGFP lines exposed to light and in the dark. r–t: Oviposition preference of *S. litura* adults for nGFP plants. u: Feeding preference of *S. litura* larvae for the 10 mg/ml FITC coated tobacco leaves exposed to light and in the dark. v–x: Feeding damage from *S. litura* larvae on transplastomic and control tobacco plants after 48 h and after 72 h. y: Feeding damage from *S. litura* larvae on the transplastomic and control tobacco plants after 15 days. z: Naturally occurring damage from lepidopterous pests on transplastomic (left) and control (right) tobacco plants in a greenhouse.

We found that *S. litura* larvae had a significant preference for transplastomic tobacco leaves that were exposed to continuous light conditions (Figure 1g–i; 6 h: *χ* ^2^ = 8.53, *P* = 0.003; 12 h: *χ* ^2^ = 11.84, *P* = 0.001; 24 h: *χ* ^2^ = 11.84, *P* = 0.001; Chi‐squared test) and that located in the darkness (Figure 1j; 6 h: *χ* ^2^ = 4.11, *P* = 0.043;12 h: *χ* ^2^ = 6.36, *P* = 0.012; 24 h: *χ* ^2^ = 10.67, *P* = 0.001; Chi‐squared test). A video record also showed that the larvae had a feeding preference for transplastomic tobacco leaves (Video S1). In addition, all of the *S. litura* larvae were trapped and died after they were released into petri dishes for 15 min (Video S2). However, the larvae showed no feeding preference for the nuclear transgenic tobacco leaves with the exception of the 6 h time point after they were transferred to the dark (*χ* ^2^ = 5.06, *P* = 0.025; Chi‐squared test) (Figure 1p,q). The larvae also had no feeding preference for tobacco leaves coated with FITC (Figures 1u and S4). The transplastomic tobacco plants were severely damaged by the *S. litura* larvae during a 48 h feeding experiment. Indeed, these plants were almost completely defoliated after 72 h (Figure 1v–x).

In the Y‐tube olfactometer test (Figures 1k and S3), we observed no chemotaxis when the *S. litura* larvae were exposed only to fresh air from both arms (Figure 1k). When either the transplastomic or control tobacco odour sources were utilized in one arm of the Y‐tube olfactometer, we observed significant chemotaxis among the larvae to the tobacco plant (pGFP: *χ* ^2^ = 3.99, *P* = 0.046; Control: *χ* ^2^ = 4.44, *P* = 0.035). However, we observed no significant difference in chemotaxis when we used the transplastomic tobacco in one arm and wild‐type tobacco in the other arm (Figure 1k). These data indicate that there is no significant chemotactic difference between the transplastomic tobacco and the non‐transgenic tobacco.

In the choice test, *S. litura* females oviposited a greater mass of eggs on the transplastomic tobacco plants relative to the control plants (Figure 1l–n; *χ* ^2^ = 40.50, *P* = 0.006). Simultaneously, a larger number of eggs were laid on the transplastomic tobacco plants than on the non‐transgenic control plants (Figure 1o; *t* = 5.97, *P* = 0.002). In contrast, no significant oviposition preference was observed for the transgenic tobacco harbouring a nuclear‐localized transgene that expresses GFP relative to the control plants (Figure 1r–t).

For the investigation in plastic pots, the transplastomic tobacco plants were fed to lepidopterous insect pests in a greenhouse. The damage was severe after 15 days (Figure 1y). At the same time, more than 90% of the transplastomic tobacco plants were severely damaged (Figures 1z and S2a). However, the adjacent non‐transgenic control plants were damaged at a rate that was <2% (Figure 1z). In addition to *S. litura*, pests collected from the damaged transplastomic tobacco plants in the greenhouse included a small number of the *Spodoptera exigua* Hübner (Lepidoptera: Noctuidae) (Figure S2b,c). GFP fluorescence present in the frass and midguts of the collected lepidopterous larvae (Figure S2d,e) indicated that those pests mainly fed on the transplastomic tobacco.

Although the crystal jelly uses fluorescence to attract prey (Steven *et al*., 2015), no one knows whether GFP provides other biological or ecological functions to jellyfish. Our findings support the idea that fluorescence from jellyfish can attract insects when they accumulate in plants. We found that *S. litura* were attracted to transplastomic tobacco that emitted green fluorescence at approximately 500 nm. Thus, combining this technology with pesticides could lead to an effective push‐pull strategy to manage and kill *S. litura*. Particular amino acid substitutions in GFP can alter the excitation and emission maxima of GFP (Verkhusha and Lukyanov, 2004). Photoresponse is widespread among insects (Casper *et al*., 2021). Thus, we can engineer fluorescent proteins that are compatible with the spectral sensitivity of specific insect photoreceptors and lure particular insects to particular plants. Recently bioluminescent plants were generated (Mitiouchkina *et al*., 2020). These approaches will lead to the development of bioluminescent and fluorescent plants that lure and trap insect pests. In summary, our findings indicate that light emitted from plants as either bioluminescence or fluorescence can contribute to ecological interactions between animals and plants and provide a novel means for monitoring and managing insects and pests. Certainly, all ethical and environmental concerns should be resolved before using this sort of biotechnology outside of the laboratory.

## Conflict of interest

The authors declare no conflict of interest.

## Author contributions

GGN designed the experiments. QLP, JZ, JJL, SYX, CCH and XXH performed the experiments. GGN, JZ and RML wrote the manuscript. MZB, JHW, GFZ, HL and GGN supervised the research. All authors participated in data interpretation.

## Supporting information


**Figure S1** Schematic representation of vectors used for transformations.
**Figure S2** Damage to the transplastomic tobacco from various *S. litura* larvae.
**Figure S3** Glass Y‐tube olfactometer used for odor selectivity testing.
**Figure S4** Feeding preference of *S. litura* larvae for tobacco leaves coated with fluorescein isothiocyanate (FITC).

**Video S1** Feeding preference of the *S. litura* larvae in the two‐choice assay on the transplastomic tobacco.

**Video S2** Trapping effect of the transplastomic tobacco leaf discs soaked in pesticides on the *S. litura* larvae.
