# Supplementary material for: Producing fluorescent plants to lure and trap insect pests
Source: Plant Biotechnol J. 2022 Jul 24;20(10):1847–9. doi: 10.1111/pbi.13887 (PMC9491452; doi:10.1111/pbi.13887)

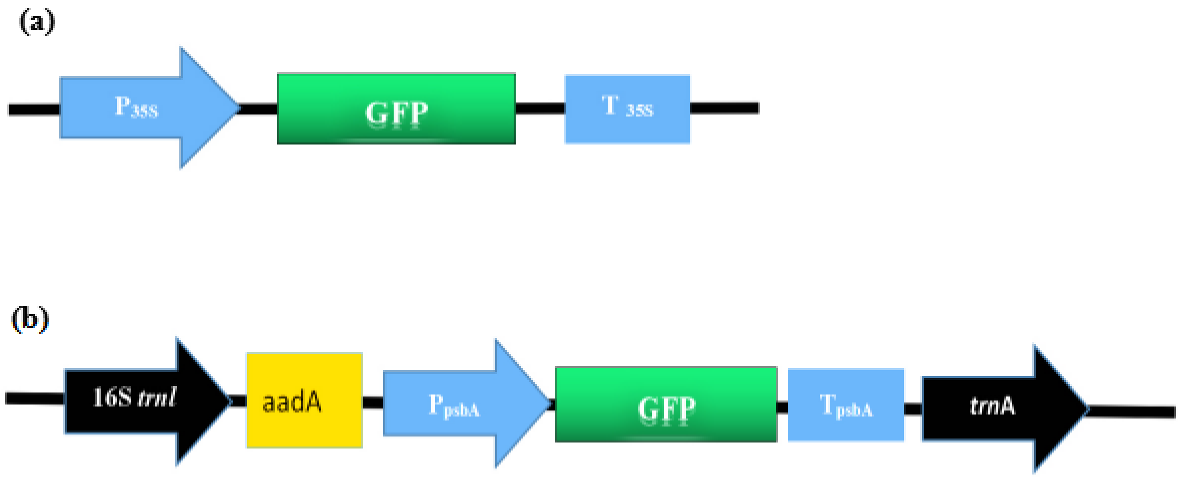

Fig.S1. Schematic representation of vectors used for transformations. (a) Vector used for the nuclear transformation. (a) Vector used for the chloroplast transformation.

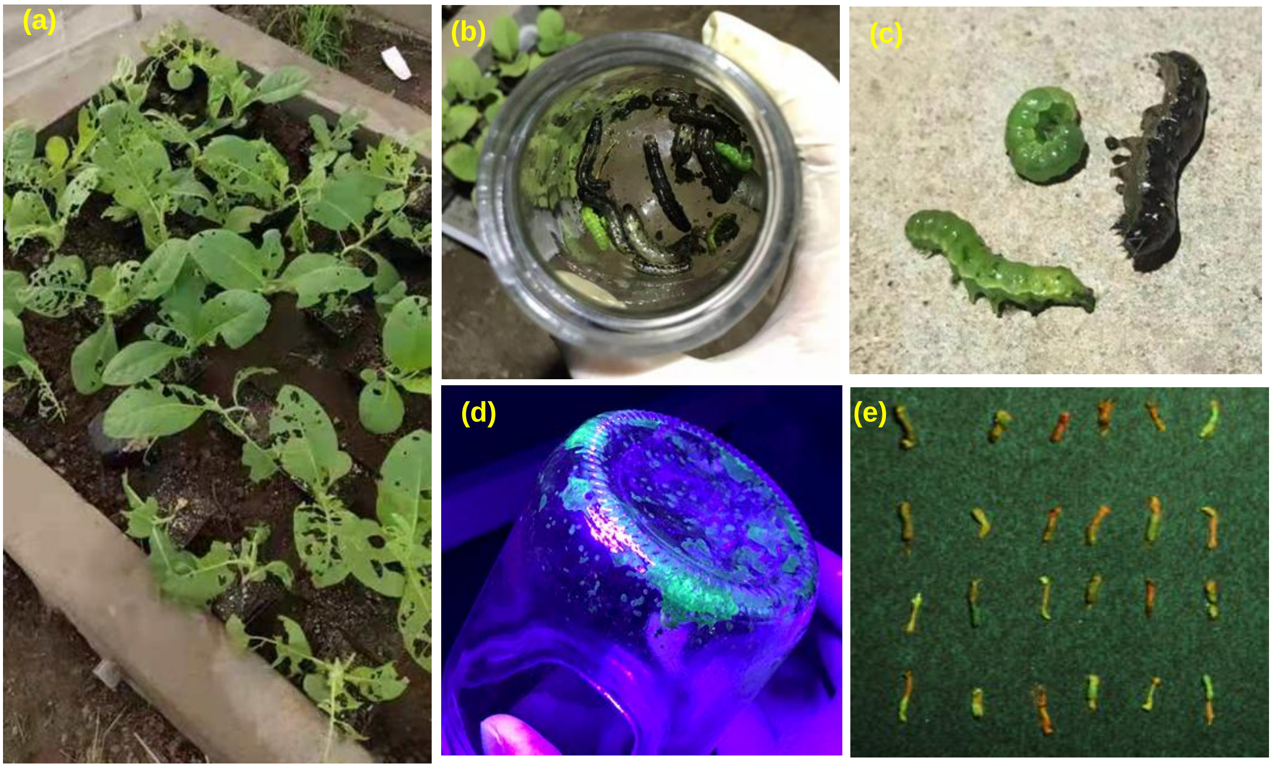

Fig.S2. Damage to the transplastomic tobacco from various S. litura larvae. (a) Damage on transplastomic tobacco plants from lepidopterous larvae. (b) Collection of naturally occurring lepidopterous larvae. (c) Identification of larvae from S. litura and S. exigua. (d) Green fluorescence of frass from the collected lepidopterous larvae. The frass was irradiated with 475 nm light, and green fluorescence was imaged.(e) Fluorescences of the midguts from the collected S. litura larvae. The midguts were irradiated with 475 nm light, and green fluorescence was imaged.

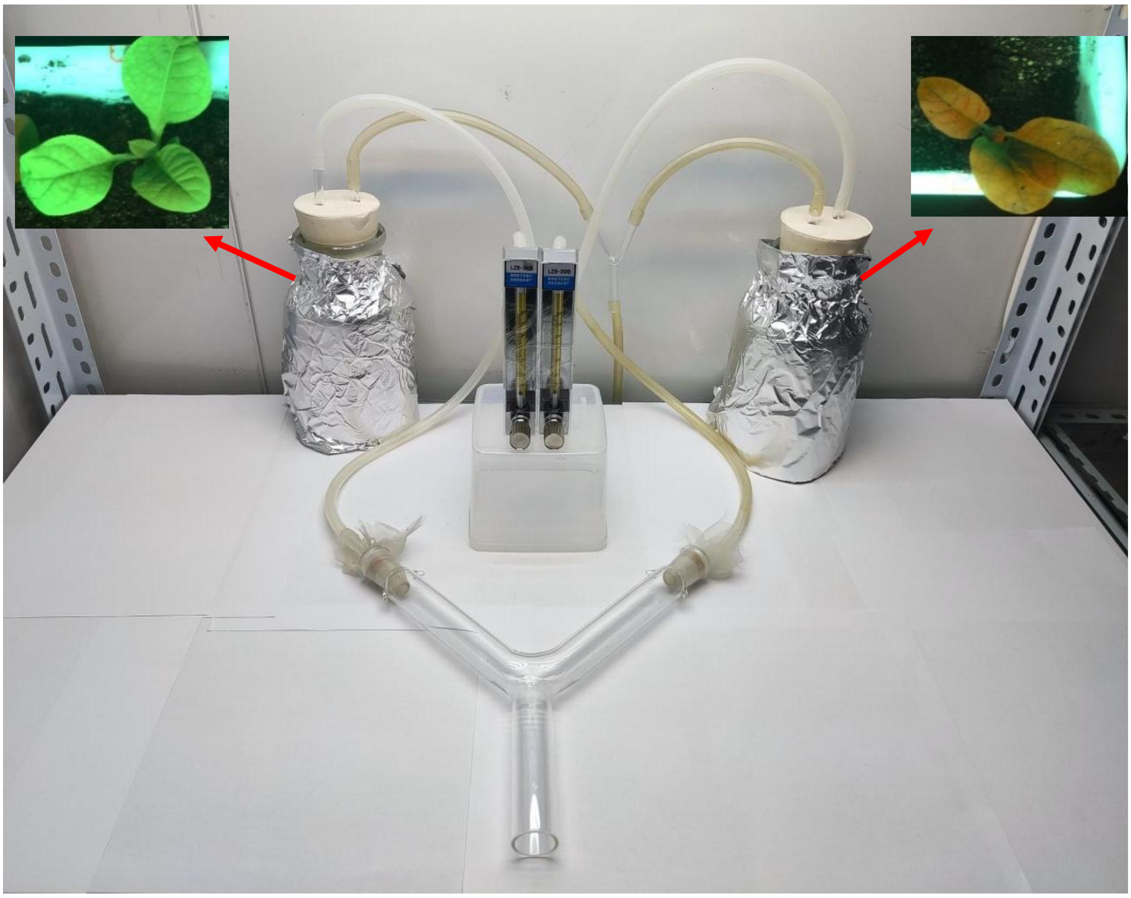

Fig.S3. Glass Y-tube olfactometer used for odor selectivity testing. The transplastomic tobacco plant that accumulates GFP in the chloroplasts emits green fluorescence. The non-transgenic tobacco plant emits red fluorescence.

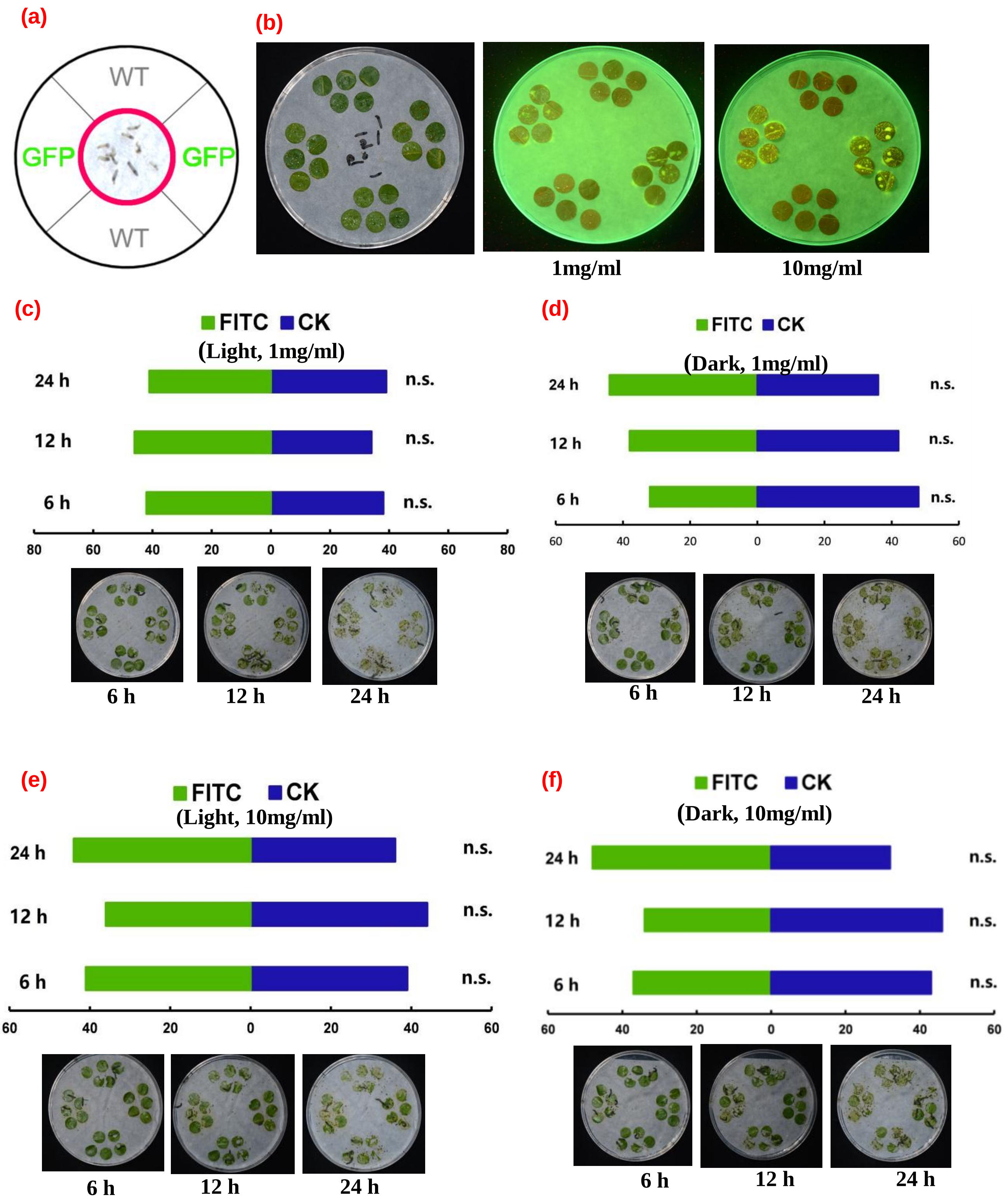

(a)
WT
GFP
GFP
WT
(b)
1mg/ml
10mg/ml
(c)
FITC CK
(Light, 1mg/ml)
24 h
12 h
6 h
n.s.
n.s.
n.s.
80 60 40 20 0 20 40 60 80
6 h
12 h
24 h
(d)
FITC CK
(Dark, 1mg/ml)
24 h
12 h
6 h
n.s.
n.s.
n.s.
60 40 20 0 20 40 60
6 h
12 h
24 h
(e)
FITC CK
(Light, 10mg/ml)
24 h
12 h
6 h
n.s.
n.s.
n.s.
60 40 20 0 20 40 60
6 h
12 h
24 h
(f)
FITC CK
(Dark, 10mg/ml)
24 h
12 h
6 h
n.s.
n.s.
n.s.
60 40 20 0 20 40 60
6 h
12 h
24 h

Fig.S4. Feeding preference of *S. litura* larvae for tobacco leaves coated with fluorescein isothiocyanate (FITC). (a) Schematic diagram of the experimental setup. (b) The experiment in white light and 475 nm light. The leaf discs were coated with 1 mg/ml and 10 mg/ml FITC. (c) Feeding preference of *S. litura* larvae for the 1 mg/ml FITC coated tobacco leaves exposed to light. (d) Feeding preference of *S. litura* larvae for 1 mg/ml FITC coated tobacco leaves in the dark. (e) Feeding preference of *S. litura* larvae for the 10 mg/ml FITC coated tobacco leaves exposed to light. (f) Feeding preference of *S. litura* larvae for the 10 mg/ml FITC coated tobacco leaves in the dark. In (c) (d), (e) and (f), n = 80 3rd *S. litura* larvae. n.s. indicates that the difference between two treatments was not statistically significant as determined by a Chi-square test.

Supplementary video1. Feeding preference of the *S. litura* larvae in the two-choice assay on the transplastomic tobacco.

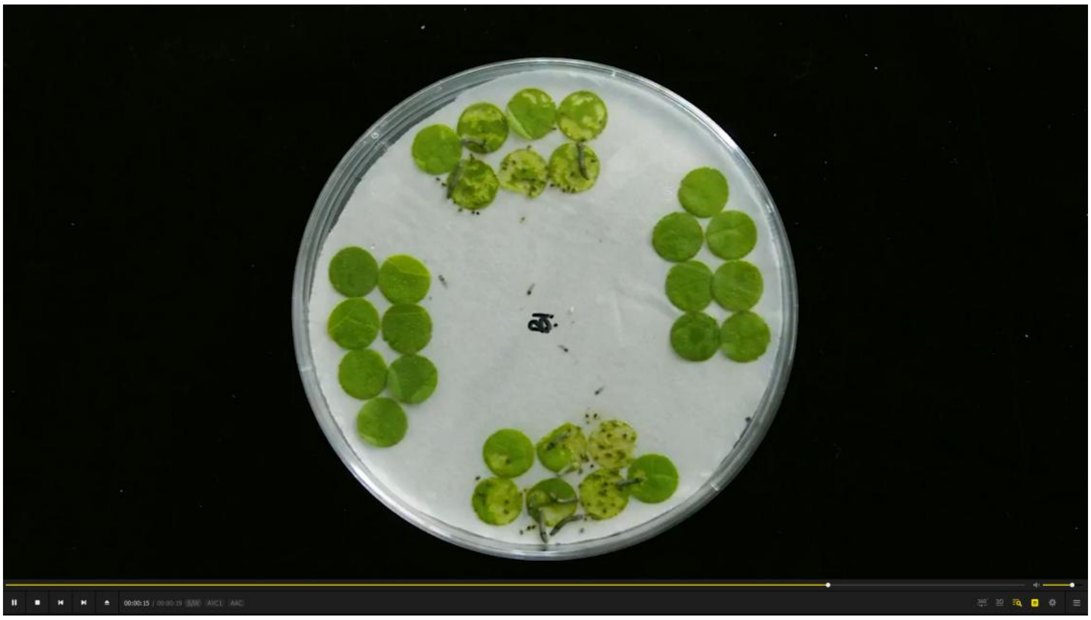

Supplementary video2. Trapping effect of the transplastomic tobacco leaf discs soaked in pesticides on the *S. litura* larvae.

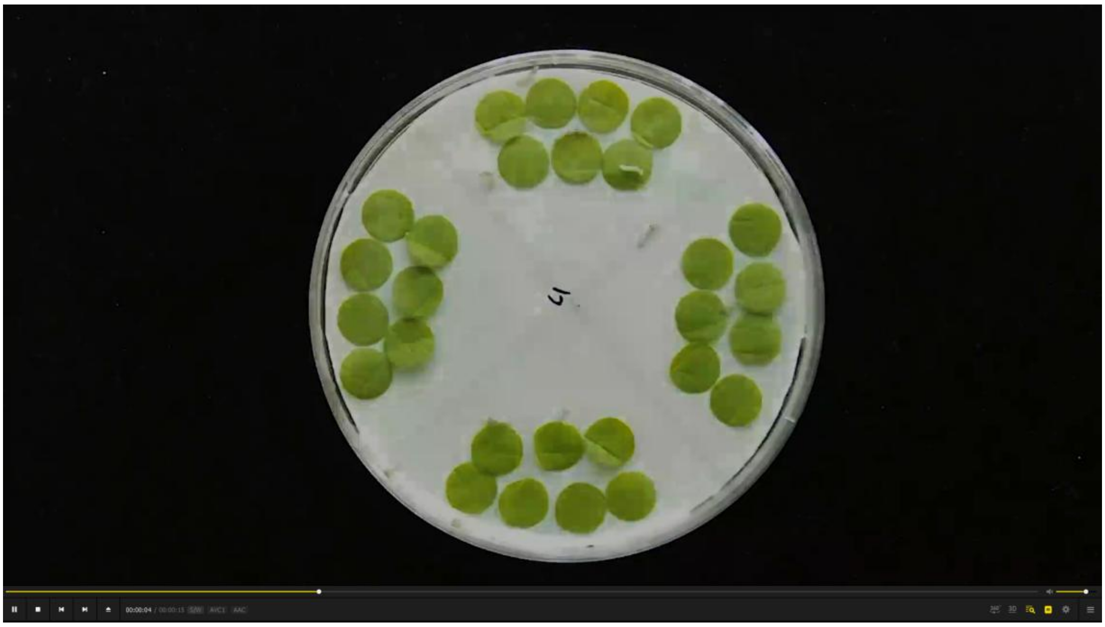

Supplement: Supplementary file 1 — Figure S1 Schematic representation of vectors used for transformations. Figure S2 Damage to the transplastomic tobacco from various S. litura larvae. Figure S3 Glass Y‐tube olfactometer used for odor selectivity testing. Figure S4 Feeding preference of S. litura larvae for tobacco leaves coated with fluorescein isothiocyanate (FITC). [file PBI-20-1847-s002.pdf]
